# The role of neoadjuvant radiochemotherapy in the management of localized high-grade soft tissue sarcoma

**DOI:** 10.1186/s13014-022-02106-2

**Published:** 2022-08-08

**Authors:** Marta Kobus, Siyer Roohani, Felix Ehret, Anne Flörcken, Jana Käthe Striefler, Franziska Brandes, Sven Märdian, Daniel Rau, Silvan Wittenberg, Robert Öllinger, David Kaul

**Affiliations:** 1 Department of Radiation Oncology, Charité – Universitätsmedizin Berlin, Corporate Member of Freie Universität Berlin and Humboldt-Universität Zu Berlin, Augustenburger Platz 1, 13353 Berlin, Germany; 2 Berlin Institute of Health at Charité – Universitätsmedizin Berlin, Charitéplatz 1, 10117 Berlin, Germany; 3 German Cancer Consortium (DKTK), partner site Berlin, and German Cancer Research Center (DKFZ), Charité – Universitätsmedizin Berlin, 69120 Heidelberg, Germany; 4 Department of Hematology, Oncology and Tumor Immunology, Charité – Universitätsmedizin Berlin, corporate member of Freie Universität Berlin and Humboldt-Universität zu Berlin, Augustenburger Platz 1, 13353 Berlin, Germany; 5 Centre for Musculoskeletal Surgery, Campus Virchow-Klinikum, Charité – Universitätsmedizin Berlin, corporate member of Freie Universität Berlin and Humboldt-Universität zu Berlin, Augustenburger Platz 1, 13353 Berlin, Germany; 6 Department of Surgery, Charité – Universitätsmedizin Berlin, Corporate Member of Freie Universität Berlin and Humboldt-Universität Zu Berlin, Augustenburger Platz 1, 13353 Berlin, Germany

**Keywords:** Soft tissue sarcoma, High-grade soft tissue sarcoma, Localized sarcoma, Sarcoma, Neoadjuvant radiotherapy, Radiochemotherapy, Prognostic factors, Univariate analysis, Multivariable analysis, Retrospective study

## Abstract

**Background:**

Standard treatment of soft tissue sarcoma (STS) of the extremities includes limb-sparing surgery combined with pre- or postoperative radiotherapy (RT). The role of perioperative chemotherapy (CTX) remains uncertain. STS patients with high-risk features for local recurrence, distant metastases, and increased mortality may require additional systemic therapy. The objective of this study was to evaluate predictors of outcome regarding local control (LC), overall survival (OS), and freedom from distant metastases (FFDM) in a large single-center cohort of patients suffering from localized high-grade STS (grade 2/3, G2/G3). Special emphasis was put on a subgroup of patients who received combined neoadjuvant radiochemotherapy (RCT).

**Methods:**

Overall, 115 adult STS patients were included in this retrospective study. The median follow-up was 34 months. Twenty-three patients (20.0%) were treated with neoadjuvant RCT, 92 (80.0%) received other therapies (adjuvant RT alone (n = 58); neoadjuvant CTX + adjuvant RT (n = 17); adjuvant RCT (n = 10), neoadjuvant RT alone (n = 7)). To assess potential prognostic factors on LC, OS, and FFDM, univariate (UVA) and multivariable (MVA) Cox proportional hazards models were applied.

**Results:**

UVA showed significantly better LC rates in the neoadjuvant RCT group (p = 0.025), with trends in MVA (p = 0.057). The 3-year LC rate was 89.7% in the neoadjuvant RCT group vs. 75.6% in the "other therapies" group. UVA also showed significantly better OS rates in the neoadjuvant RCT group (p = 0.049), however, this was not confirmed in MVA (p = 0.205), the 3-year OS rate was 85.8% for patients treated with neoadjuvant RCT compared to 73.5% in the "other therapies" group. UVA showed significantly better FFDM rates in (p = 0.018) and a trend towards better FFDM rates in MVA (p = 0.059). The 3-year FFDM rate was 89.7% for patients treated with neoadjuvant RCT compared to 65.9% in the "other therapies" group. In the subgroup of patients with G3 STS, neoadjuvant RCT was a significant positive predictor of LC and FFDM in MVA (p = 0.047, p = 0.027) but not for OS. Overall grade 3 and 4 toxicities were significantly higher (p = 0.019) in the neoadjuvant RCT group and occurred in 73.9% vs. 38.0% in patients receiving other therapies.

**Conclusions:**

The results suggest that neoadjuvant RCT might improve LC and FFDM in patients with localized G3 STS while also being associated with increased acute complication rates. Further prospective research is warranted to confirm these findings.

**Supplementary Information:**

The online version contains supplementary material available at 10.1186/s13014-022-02106-2.

## Highlights

- Neoadjuvant radiochemotherapy may achieve better local control in high-grade (G3) soft tissue sarcoma than other therapies.
- Neoadjuvant radiochemotherapy may achieve better freedom from distant metastases in high-grade (G3) soft tissue sarcoma than other therapies.
- Neoadjuvant radiochemotherapy is associated with higher rates of acute major complications than other therapies.

## Introduction

Soft tissue sarcomas (STS) are a rare and heterogeneous group of malignant tumors accounting for less than 1% of all solid malignancies in adults [1]. The diagnosis is challenging due to the histological heterogeneity—more than 100 subtypes of STS have been described [2]. Histological tumor grading is applied according to the National Cancer Institute or the French Fédération Nationale des Centres de Lutte Contre le Cancer (FNCLCC) [3, 4]. While STS can arise in virtually all anatomic locations, the extremities are the most frequent (43%), followed by visceral (19%) and retroperitoneal sites (15%) [5].

Therapy of STS should preferably be carried out at specialized sarcoma centers [6–8]. The multimodal therapy of localized high-grade STS includes surgery and pre- or postoperative radiotherapy (RT) [9, 10]. The role of chemotherapy (CTX) and regional hyperthermia remains controversial [11]. Most patients with localized STS show good long-term outcomes with wide excision and RT [12, 13]. However, a large proportion of patients carry unfavorable features (high-risk features) for local recurrence (LR), including positive surgical margins, presentation with locally recurrent disease or older age [14, 15]. Moreover, high-risk features for distant metastases (DM) and shorter overall survival (OS) are high-grade STS, large tumor size, and certain histological subtypes [14, 16–18].

The significance of concomitant CTX added to neoadjuvant RT in the management of STS remains unclear. Combined neoadjuvant radiochemotherapy (RCT) may increase the local effect of RT through radiosensitization and provide control of potential micrometastases [19]. However, to date, no large, randomized trial has compared neoadjuvant RCT to RT alone. We evaluated predictors of outcome for local control (LC), OS, and freedom from distant metastases (FFDM) in a large single-center cohort of patients with the initial diagnosis of localized high-grade STS (grade 2/3, G2/G3). We put special emphasis on comparing the subgroup of patients who received neoadjuvant RCT.

## Materials and methods

This single-center retrospective study included adult patients initially diagnosed with localized high-grade (G2/3) STS according to the FNCLCC between 2004 and 2020. The inclusion criteria were: primary diagnosis of histopathologically confirmed and resected high-grade STS, neoadjuvant or adjuvant RT, CTX or RCT of the STS, tumor located in the extremities, pelvis, head and neck, trunk wall, retroperitoneum or intraabdominally. Exclusion criteria were metastatic or recurrent disease at the time of diagnosis, age < 18 years, the most common STS of childhood and adolescence (rhabdomyosarcoma, Ewing sarcoma) and sarcoma-like lesions (desmoid fibromatosis or dermatofibrosarcoma protuberans).

We reviewed the medical records, pathological, and radiological reports of eligible patients. The study was approved by the institutional review board (EA1/163/21). Endpoints included LC, OS, FFDM, and acute toxicities. Toxicity was evaluated according to the Common Terminology Criteria for Adverse Events version 5.0 and classified as acute if it occurred within three months or late if it occurred after three months after treatment completion [20]. Major complications were defined as grade ≥ 3 [20].

The prescribed radiation dose for a large proportion of patients was 1.8 to 50.4 Gy with a simultaneous integrated boost (SIB) of 2.0 to 56 Gy. The planned target volume included the macroscopic tumor + 3 cm transversal and 5 cm longitudinal safety margins. The additional SIB dose of 2.0 to 56 Gy was applied to the macroscopic tumor volume alone visible on planning computed tomography and t2-weighted magnetic resonance imaging.

Statistical analysis was performed using IBM SPSS Statistics 27. T-tests were two-sided. A p-value of ≤ 0.05 was considered statistically significant. A p-value of > 0.05–≤ 0.1 was considered a trend. Group comparisons of continuous variables were done using the t-test and the Mann–Whitney U test. Dichotomization of continuous variables (Karnofsky Performance Status (KPS), age, and tumor size) was done using the median of the respective variable. Group comparisons of categorical variables was made using the Chi-square test. For time-to-event variables, the Kaplan–Meier estimate was used. Univariate and multivariable Cox proportional hazards models were performed to analyze the influence of various factors on LC, OS, and FFDM. LC, OS, and FFDM were calculated from the date of initial surgery. We incorporated the variables with significant outcomes from univariate analysis (UVA) into the multivariable analysis (MVA). In LC and FFDM analysis, patients were censored on the date of death or last contact.

## Results

### Patients, tumor, and treatment characteristics

Patient’, tumor-, and treatment characteristics are shown in Table 1. Out of 204 initially identified STS patients, 115 patients met the eligibility criteria and were retained for analysis. The median follow-up time was 34 months (range, 3–206 months).

**Table 1:** Baseline characteristics

Characteristic	Total (N = 115)	(%)	Neoadjuvant RCT (N = 23)	(%)	Other therapies (N = 92)	(%)
Sex						
Male	62	53.9	13	56.5	49	53.3
Female	53	46.1	10	43.5	43	46.7
Mean age, years (range)	59.27 (20–95)		52.7 (23–74)		60.91 (20–95)	
Karnofsky Performance Status						
40	1	0.9	0	0	1	1.1
50	0	0	0	0	0	0
60	4	3.5	0	0	4	4.3
70	4	3.5	0	0	4	4.3
80	36	31.3	5	21.7	31	33.7
90	56	48.7	13	56.5	43	46.7
100	13	11.3	5	21.7	8	8.7
n/a	1	0.9	0	0	1	1.1
Anatomic location						
Upper extremity	10	8.7	0	0	10	10.9
Lower extremity	66	57.4	18	78.3	48	52.2
Pelvis	6	5.2	3	13	3	3.3
Head/neck	6	5.2	0	0	6	6.5
Trunk wall	15	13	1	4.3	14	15.2
Retroperitoneal	8	7	1	4.3	7	7.6
Intra-abdominal	4	3.5	0	0	4	4.3
Sarcoma Subtype						
Liposarcoma/Myxoid Liposarcoma	25	21.7	1	4.3	24	26.1
Myxofibrosarcoma	26	22.6	6	26.1	20	21.7
Undifferentiated pleomorphic sarcoma	21	18.3	5	21.7	16	17.4
Synovial cell sarcoma	12	10.4	5	21.7	7	7.6
Malignant peripheral nerve sheath tumor	8	7	1	4.3	7	7.6
Leiomyosarcoma	9	7.8	1	4.3	8	8.7
Angiosarcoma	5	4.3	2	8.7	3	3.3
Spindle cell sarcoma	3	2.6	0	0	3	3.3
Fibrosarcoma	1	0.9	0	0	1	1.1
Giant cell sarcoma	1	0.9	0	0	1	1.1
Small cell/Clear cell sarcoma	2	1.7	1	4.3	1	1.1
Epitheloid sarcoma	1	0.9	1	4.3	0	0
Myxoinflammatory fibroblastic sarcoma	1	0.9	0	0	1	1.1
Mean tumor size, cm (range)	10.63 (1.5–41.6)		10.35 (3.5–16)		10.71 (1.5–41.6)	
Tumor depth						
Superficial	14	12.2	2	8.7	12	13
Deep	85	73.9	20	87	65	70.7
n/a	16	13.9	1	4.3	14	15.2
Grade						
G2	53	46.1	8	34.8	45	48.9
G3	62	53.9	15	65.2	47	51.1
Resection margin						
R0	92	80	21	91.3	71	77.2
R1	10	8.7	1	4.3	9	9.8
R2	2	1.7	0	0	2	2.2
n/a	11	9.6	1	4.3	10	10.9
Mean single radiation dose, Gy (range)	2.1 (1.2–5.0)		1.9 (1.6–2.15)		2.13 (1.2–5.0)	
Mean total radiation dose, Gy (range)	58.4 (25.0–75.7)		54.1 (48.8–60.2)		59.5 (25.0–75.7)	
Targeted therapy	1	0.9	0	0	1	1.1
Hyperthermia	22	19.1	2	8.7	20	21.7
Local recurrence/progress	27	23.5	2	8.7	25	27.2
Therapy local recurrence						
Resection	20	17.4	2	8.7	18	19.6
Radiotherapy	4	3.5	0	0	5	5.4
Chemotherapy	11	9.6	0	0	11	12
Targeted Therapy	2	1.7	0	0	2	2.2
Hyperthermia	3	2.6	0	0	3	3.3
Distant metastases	42	36.5	6	26.1	36	39.1
Lungs	33	28.7	5	21.7	28	30.4
Bones	3	2.6	0	0	3	3.3
Liver	4	3.5	0	0	4	4.3
Lymph nodes	2	1.7	1	4.3	1	1.1
Other	7	6.1	0	0	7	7.6
Therapy distant metastases						
Resection	20	17.4	5	21.7	15	16.3
Radiotherapy	9	7.8	1	4.3	8	8.7
Chemotherapy	16	13.9	1	4.3	15	16.3
Targeted Therapy	1	0.9	0	0	1	1.1
Hyperthermia	2	1.7	0	0	2	2.2
Death	37	32.2	6	26.1	31	33.7

*n/a* not available

Patients treated with neoadjuvant RCT were younger than patients who received other therapies (mean age 52.7 vs. 60.9 years) and had larger median tumor diameters (11.0 cm vs. 8.0cm in the "other therapies" group respectively). The mean total dose of RT was higher in the "other therapies" group than in the neoadjuvant RCT group with 59.5 Gy and 54.1 Gy, respectively.

In the neoadjuvant RCT group (n = 23), most patients (n = 21, 91.3%) received a combination of doxorubicin and ifosfamide. The most common regimen (n = 14, 60.9%) consisted of three initial cycles of doxorubicin (60 mg/m^2^/d for d1–d2) plus ifosfamide (3000 mg/m^2^/d for d1–d3) intravenously followed by RT (50.4/56 Gy in 1.8/2 Gy fractions with a SIB) and two concomitant cycles of ifosfamide, followed by a final cycle of doxorubicin and ifosfamide. Two patients from the neoadjuvant RCT group did not receive anthracyclines due to cardiac comorbidities. The 92 patients receiving other therapies can further be subdivided into adjuvant RT (n = 58), neoadjuvant CTX plus adjuvant RT (n = 17), adjuvant RCT (n = 10) and neoadjuvant RT (n = 7).

### Oncological outcomes

R0 resection was achieved in 71 of 92 (77.2%) patients receiving other therapies and 21 of 23 (91.3%) patients from the RCT group, with no statistically significant difference (p = 0.317).

After neoadjuvant RCT and resection, histological assessment found seven of 23 (30.4%) patients to not have any viable appearing tumor cells. Three patients (13%) had single vital tumor cells or one vital cell cluster < 0.5 cm, five (21.7%) had vital tumor tissue of less than 10%, two (8.7%) had viable tumor tissue of between 10 and 50% and four (17.4%) had vital tumor tissue of more than 50% at the time of surgery. All patients treated with neoadjuvant RCT showed some degree of histological tumor cell death [21].

#### Local control

Table 2 shows the UVA and MVA for LC. Median time to recurrence in the overall cohort was 206 months, with a 3-year LC rate of 89.7% in the neoadjuvant RCT group versus 75.6% in the "other therapies" group. LC rates were significantly higher in the neoadjuvant RCT group in UVA and there was a trend towards higher rates on MVA (p = 0.025 and p = 0.057). In G3 STS, neoadjuvant RCT was a significant factor for LC in UVA and MVA (p = 0.022 and p = 0.047). In the entire cohort, negative surgical margins (p < 0.001 and p = 0.012 on UVA and MVA) and extremity location of the tumor (p = 0.001 and p = 0.007 on UVA and MVA) were associated with a better LC.

**Table 2:** Univariate and multivariable analysis of LC

Variable	All (N = 115)				G2 sarcoma (N = 53)				G3 sarcoma (N = 62)			
	Univariate analysis		Multivariable analysis		Univariate analysis		Multivariable analysis		Univariate analysis		Multivariable analysis	
	HR (95% CI)	p-Value	HR (95% CI)	p-Value	HR (95% CI)	p-Value	HR (95% CI)	p-Value	HR (95% CI)	p-Value	HR (95% CI)	p-Value
Age (in years)												
< 61	Ref				Ref				Ref			
≥ 61	.881 (.407–1.909)	.749			.584 (.155–2.202)	.427			1.069 (.380–3.009)	.899		
Sex												
Male	Ref				Ref				Ref			
Female	.888 (.407–1.934)	.764			.386 (.113–1.318)	.129			1.896 (.686–5.244)	.218		
KPS												
< 90	Ref				Ref				Ref		Ref	
≥ 90	.595 (.275–1.289)	.188			1.249 (.365–4.274)	.724			.330 (.112-.968)	.044*	.622 (.203–1.903)	.405
Location												
Other	Ref		Ref		Ref		Ref		Ref			
Extremity	.262 (.120-.572)	.001*	.300 (.124-.724)	.007*	.151 (.040-.576)	.006*	.293 (.066–1.302)	.107	.363 (.128–1.029)	.057		
Tumor size (in cm)												
< 8.8	Ref				Ref				Ref			
≥ 8.8	1.006 (.459–2.208)	.987			1.873 (.571–6.142)	.300			.580 (.201–1.675)	.314		
Resection margin												
R0	Ref		Ref		Ref		Ref		Ref			
R1/2	6.457 (2.738–15.228)	< .001*	3.303 (1.300–8.396)	.012*	13.434 (3.433–52.567)	< .001*	7.469 (1.649–33.828)	.009*	2.00 (.258–15.479)	.507		
n/a	1.017 (.234–4.426)	.982	.411 (.086–1.972)	.266	1.640 (.170–15.817)	.669	.950 (.090–10.006)	.966	1.133 (.148–8.685)	.905		
Therapy												
Other therapy	Ref		Ref		Ref				Ref		Ref	
Neoadjuvant RCT	.191 (.045-.815)	.025*	.236 (.053–1.044)	.057	.502 (.064–3.935)	.512			.091 (.012-.708)	.022*	.116 (.014-.974)	.047*
Hyperthermia												
No	Ref				Ref				Ref			
Yes	2.245 (.888–5.674)	.087			2.982 (.780–11.396)	.110			2.071 (.554–7.748)	.279		

*n/a* not available

#### Overall survival

Table 3 shows the UVA and MVA for OS. The median OS was 113 months after diagnosis. In the neoadjuvant RCT group, the 3-year OS rates were 85.8% compared to 73.5% in the "other therapies" group. In UVA, OS differed significantly between both groups (p = 0.049), but the finding was not confirmed in MVA (p = 0.205). However, there was a trend towards a higher OS rate among patients with G3 STS treated with neoadjuvant RCT in MVA (p = 0.068). Although R0 resection margin did show a significantly increased survival rate in UVA compared to R1 or R2 (p = 0.008), MVA did not confirm this result (p = 0.092). KPS ≥ 90% was shown to be a positive predictor of survival in both UVA and MVA (p = 0.006 and p = 0.046). Moreover, the tumor location in the extremity also correlated with better OS in G2 STS (p = 0.015 and p = 0.029 in UVA and MVA). ﻿﻿In an alternative analysis where only patients with neoadjuvant RCT vs. neoadjuvant RT were included, a significantly better OS in favor of neoadjuvant RCT was observed in UVA and MVA in G3 tumors (p = 0.001 and p = 0.010, Additional file 1: Table 1﻿).

**Table 3:** Univariate and multivariable analysis of OS

Variable	All (N = 115)				G2 sarcoma (N = 53)				G3 sarcoma (N = 62)			
	Univariate analysis		Multivariable analysis		Univariate analysis		Multivariable analysis		Univariate analysis		Multivariable analysis	
	HR (95% CI)	p-Value	HR (95% CI)	p-Value	HR (95% CI)	p-Value	HR (95% CI)	p-Value	HR (95% CI)	p-Value	HR (95% CI)	p-Value
Age (in years)												
< 61	Ref				Ref				Ref			
≥ 61	1.951 (.991–3.842)	.053			2.482 (.807–7.636)	.113			1.446 (.617–3.387)	.396		
Sex												
Male	Ref				Ref	.303			Ref			
Female	.853 (.442–1.646)	.636			.563 (.189–1.680)				1.271 (.554–2.915)	.572		
KPS												
< 90	Ref		Ref		Ref				Ref		Ref	
≥ 90	.386 (.197-.756)	.006*	.485 (.238-.988)	.046*	.444 (.144–1.365)	.156			.372 (.162-.858)	.020*	.577 (.232–1.432)	.236
Location												
Other	Ref				Ref		Ref		Ref			
Extremity	.532 (.271–1.042)	.066			.244 (.078-.761)	.015*	.240 (.066-.867)	.029*	.774 (.305–1.963)	.590		
Tumor size (in cm)												
< 8.8	Ref				Ref				Ref			
≥ 8.8	1.356 (.707–2.603)	.359			1.369 (.459–4.079)	.573			1.246 (.545–2.852)	.602		
Resection margin												
R0	Ref		Ref		Ref		Ref		Ref			
R1/2	3.090 (1.336–7.146)	.008*	2.114 (.884–5.058)	.092	4.648 (1.429–15.113)	.011*	2.252 (.620–8.186)	.218	2.137 (.480–9.512)	.319		
n/a	.577 (.137–2.424)	.453	.477 (.113–2.010)	.313	-	-			1.561 (.362–6.731)	.550		
Therapy												
Other therapy	Ref		Ref		Ref				Ref		Ref	
Neoadjuvant RCT	.405 (.165-.994)	.049*	.544 (.212–1.394)	.205	.830 (.182–3.793)	.810			.234 (.075-.729)	.012*	.318 (.093–1.090)	.068
Hyperthermia												
No	Ref				Ref				Ref			
Yes	1.130 (.433–2.946)	.803			1.353 (.295–6.194)	.697			1.029 (.296–3.573)	.965		

*n/a* not available

* = *p* < 0.05, statistically significant

#### Freedom from distant metastases

Table 4 shows UVA and MVA for FFDM. The median time to metastasis was 105 months with a 3-year FFDM rate of 89.7% in the neoadjuvant RCT group compared to 65.9% in the "other therapies" group. In UVA of the entire cohort, FFDM differed significantly between the neoadjuvant RCT and "other therapies" group in favor of neoadjuvant RCT (p = 0.018). In the MVA there was a trend towards higher FFDM rate among patients treated with neoadjuvant RCT (p = 0.059). Similar to the LC rates, the FFDM for the subgroup of G3 sarcomas again indicated a positive effect of neoadjuvant RCT with significant findings for both, UVA and MVA (p = 0.002 and p = 0.027). Moreover, a higher KPS was a positive prognostic factor for FFDM in UVA (p = 0.021) and showed a trend in MVA (p = 0.088). An alternative UVA and MVA comparing neoadjuvant RCT to neoadjuvant RT alone revealed a significant and favorable contribution of neoadjuvant RCT for the FFDM rate in G3 STS (p < 0.001 in both UVA and MVA, Additional file 2: Table 2).

**Table 4:** Univariate and multivariable analysis of FFDM

Variable	All (N = 115)				G2 sarcoma (N = 53)				G3 sarcoma (N = 62)			
	Univariate analysis		Multivariable analysis		Univariate analysis		Multivariable analysis		Univariate analysis		Multivariable analysis	
	HR (95% CI)	p-Value	HR (95% CI)	p-Value	HR (95% CI)	p-Value	HR (95% CI)	p-Value	HR (95% CI)	p-Value	HR (95% CI)	p-Value
Age (in years)												
< 61	Ref				Ref				Ref			
≥ 61	1.137 (.614–2.105)	.683			.732 (.186–2.883)	.655			1.038 (.507–2.125)	.918		
Sex												
Male	Ref				Ref				Ref			
Female	.744 (.399–1.387)	.352			1.112 (.323–3.823)	.866			.885 (.406–1.926)	.757		
KPS												
< 90	Ref		Ref		Ref				Ref		Ref	
≥ 90	.476 (.253-.895)	.021*	.569 (.298–1.087)	.088	2.361 (.488–11.419)	.285			.306 (.143-.652)	.002*	.496 (.219–1.135)	.093
Location												
Other	Ref				Ref				Ref			
Extremity	.818 (.415–1.612)	.561			1.893 (.399–8.971)	.421			.535 (.250–1.143)	.106		
Tumor size (in cm)												
< 8.8	Ref				Ref				Ref			
≥ 8.8	1.282 (.686–2.394)	.436			.766 (.197–2.977)	.700			1.284 (.609–2.704)	.511		
Resection margin												
R0	Ref				Ref				Ref			
R1/2	1.443 (.510–4.086)	.489			2.092 (.417–10.490)	.370			1.780 (.415–7.644)	.438		
n/a	.624 (.188–2.072)	.441			-	-			1.724 (.519–5.727)	.374		
Therapy												
Other therapy	Ref		Ref		Ref				Ref		Ref	
Neoadjuvant RCT	.344 (.141-.836)	.018*	.415 (.166–1.036)	.059	.965 (.195–4.767)	.965			.182 (.061-.540)	.002*	.267 (.083-.862)	.027*
Hyperthermia												
No	Ref				Ref				Ref			
Yes	1.890 (.857–4.171	.115			2.510 (.514–12.258)	.256			1.800 (.714–4.541)	.213		

*n/a* not available

### Toxicity

Data on major acute toxicity (grade ≥ 3) are shown in Table 5. No treatment-related death was observed. Data for toxicity were missing for 16 (13.9%) patients. Data on late toxicity were only available in 21 (18.2%) patients. Therefore, no detailed analysis of late toxicity was performed. Overall, major toxicity (grade 3 or 4) was significantly higher with neoadjuvant RCT compared to other therapies (73.9% vs. 38.0%, p = 0.019) while major hematological toxicity occurred in 12 patients (52.2%) from the neoadjuvant RCT group vs. 17 patients (18.5%) from the “other therapies” group (p < 0.001). Moreover, the rate of grade 4 febrile neutropenia requiring hospitalization was significantly higher under neoadjuvant RCT compared to other therapies (39.1% vs. 6.5%, p < 0.001). Non-hematological toxicity was limited and without statistically significant differences among both groups. Local toxicity with major wound complications (grade 3 or 4) were seen in 26.1% and 16.3% of patients under neoadjuvant RCT and other therapies, respectively (p = 0.50).

**Table 5:** Acute toxicity (greater than grade 2) according to the Common Terminology Criteria for Adverse Events version 5.0.

Toxicity	Neoadjuvant RCT n (%)		Other therapies n (%)	
	Grade 3	Grade 4	Grade 3	Grade 4
Hematological				
Anemia	4 (17.4)	0 (0)	9 (9.8)	1 (1.1)
Leukopenia	3 (13)	3 (13)	2 (2.2)	3 (3.3)
Lymphocytopenia	7 (30)	1 (4.3)	7 (7.6)	1 (1.1)
Neutropenia	0 (0)	9 (39.1)	1 (1.1)	6 (6.5)
Thrombocytopenia	3 (13)	1 (4.3)	1 (1.1)	1 (1.1)
Overall hematological	12 (52.2%) (Grade 3 + 4)		17 (18.5%) (Grade 3 + 4)	
Fatigue	3 (13)	0 (0)	4 (4.3)	0 (0)
Gastrointestinal				
Nausea	1 (4.3)	0 (0)	5 (5.4)	0 (0)
Dysgeusia	0 (0)	0 (0)	1 (1.1)	0 (0)
Dysphagia	0 (0)	0 (0)	1 (1.1)	0 (0)
Dermatological				
Erythema	0 (0)	0 (0)	3 (3.3)	0 (0)
Radiodermatitis	2 (8.7)	0 (0)	2 (2.2)	0 (0)
Hyperpigmentation	0 (0)	0 (0)	3 (3.3)	0 (0)
Epitheliolysis	0 (0)	0 (0)	2 (2.2)	0 (0)
Lymphedema	0 (0)	0 (0)	3 (3.3)	0 (0)
Inflammation				
Mucositis	1 (4.3)	0 (0)	1 (1.1)	1 (1.1)
Stomatitis	2 (8.7)	0 (0)	0 (0)	0 (0)
Neurological				
Psychosis	0 (0)	0 (0)	1 (1.1)	0 (0)
Wound complication	6 (26.1)	0 (0)	10 (10.9)	5 (5.4)
Other	1 (4.3)	0 (0)	1 (1.1)	1 (0)

No grade 5 toxicity was observed

## Discussion

Herein, we report our single institutional experience on therapeutic outcomes of neoadjuvant RCT compared to other therapy modalities for localized high-grade STS.

Optimal management of localized high-grade STS is challenging and subject of ongoing debates. Standard treatment for localized G2 or G3 STS of the extremities includes wide excision and preoperative RT [9, 10, 22]. However, on the subject of adding systemic therapy for patients with high-risk features guidelines still recommend individual assessment in multidisciplinary tumor boards due to a lack of phase III data comparing RCT to RT alone [9, 10, 14–18]. Taken together, patients with high-risk features constitute a substantial proportion of STS cases and therefore may require detailed prognostic evaluation and additional therapy [18, 23]. Although RCT is not considered standard of care, many rationales exist for adding CTX to RT for high-risk STS patients: intensification of treatment may decrease LR (1); lower risk of distant recurrence (DR) and improve OS (2); improve symptom control (pain relief) (3); therapeutic effects by CTX including radiosensitization allowing reduction of RT doses, thus lowering wound complication rates (4) [9, 10, 23–26].

Generally, our data support these rationales by showing favorable 3-year LC rates of 89.7% compared to 75.6% in the "other therapies" group. Particularly in patients with G3 sarcomas, RCT appears to have positive contributions. The high LC rates support previously published data including the pilot phase II study on neoadjuvant RCT by DeLaney et al. conducted at the Massachusetts General Hospital (MGH) in 2003 [19, 27–29]. With an RCT regimen consisting of 44 Gy of normofractionated preoperative RT with interdigitated CTX (mesna, Adriamycin (doxorubicin), ifosfamide and dacarbazine (MAID protocol)), the authors achieved remarkable LC rates in a total of 48 patients (5-year LC: 92%). One possible explanation for the better LC through neoadjuvant RCT in both, the present trial and the MGH trial, lies in the tendency of a higher rate of R0 resections observed in the neoadjuvant RCT groups (91.3% (n = 21) in the present trial and 85.4% (n = 41) in the MGH trial) compared to other therapies (77.2% in the present trial and 81.25% (n = 39) in the MGH trial), although the difference was not significant in the present trial [29]. A subsequent trial at MGH, also applying the MAID regimen, found comparable results [19]. Furthermore, a more recent retrospective single-center analysis on neoadjuvant RCT by Byun et al. observed similar results with successful resection rates (72.4% R0 (n = 21), 27.6% close margin (< 1 mm, n = 8), no cases of R1 resection) and subsequently good LC rates (86.7% at 5 years) [30]. Positive surgical margins are an established risk factor for LR and are therefore considered a high-risk feature [14, 15, 31–33]. Accordingly, R1 or R2 resection had a significant negative prognostic value on LC in the entire cohort in the present study (p = 0.012 in MVA). Moreover, once patients present with recurrent disease their mortality rates increase substantially [14, 34].

Our results confirm previous data and support the combination of CTX and RT in the preoperative setting to improve the chance of R0 resection, thereby lowering the risk of LR and mortality [23, 35]. However, conducting large, well-designed phase III randomized trials for rare malignancies remains challenging and cost-intensive.

With regards to survival, we found neoadjuvant RCT to be supportive by gaining 12.3% of OS at 3 years (85.8% compared to 73.5% with other therapies). A positive effect was also shown in UVA of predictive factors for OS in G3 sarcomas (p = 0.012) and showed a trend in MVA (p = 0.068). Similarly, DeLaney et al. found a substantial OS increase of 29% at 3-years in patients treated with neoadjuvant RCT compared to historical controls treated with RT and resection alone (87% vs. 58% (n = 48 in both groups), p = 0.0003). Even seven years after treatment, the survival benefit of the intensive RCT regimen in the MGH study sustained (36). The subsequent MGH trial applying the MAID regimen CTX together with preoperative RT confirmed the favorable results [19]. Interestingly, in the Radiation Therapy Oncology Group (RTOG) 9514 trial, where a treatment regimen very similar to DeLaney et al. was used on 64 patients, the OS was poorer (3-year OS of 75.1%) [29, 36]. The median tumor size and histological subtypes were balanced in both trials [29, 36]. The increase in mortality compared to our study and the MGH trial was most likely associated with the higher proportion of G3 sarcomas (80% (n = 51) in RTOG9514 vs. 48% (n = 23) in the MGH trial vs. 65.2% (n = 15) in our study).

A different systemic approach to be mentioned is the addition of radiosensitizing agents to RT of STS such as the poly ADP ribose polymerase inhibitor olaparib. Preliminary data from an ongoing phase Ib trial testing olaparib with normofractionated external beam RT on a cohort of 41 unresectable STS have shown promising tumor responses with favorable toxicity profiles at the six months interim analysis [37]. Although these agents have been used for unresectable STS, the first results warrant further trials testing these agents [37, 38].

High histological grade and large tumor size are independent adverse prognostic factors for OS and may therefore also be considered high-risk features requiring additional measures such as adding CTX to RT to improve patients' outcomes [14, 18, 23, 39]. We found a promising 3-year FFDM rate of 89.7% in the neoadjuvant RCT group (vs. 65.9% by other therapies) with a significant finding in the UVA (p = 0.018) and a trend in MVA (p = 0.059) of the cox regression analysis. Particularly in the G3 sarcoma subgroup, neoadjuvant RCT significantly reduced the hazard ratio for distant metastasis (p = 0.002 in UVA, p = 0.027 in MVA). This data suggests promising effects of preoperative RCT and warrants further, comparative studies with a well-matched control arm treated with preoperative RT alone.

The foremost concerns of adding CTX to preoperative RT are increased systemic toxicity by CTX and higher wound complication rates.

After applying a median total dose of 56 Gy, in a mean single dose of 1.9 Gy/fraction in the RCT group and 60.2 Gy, 2 Gy/fraction in the "other therapies" group, we found no significant differences in wound complication rates among both groups (26.1% in preoperative RCT vs. 16.3% (p = 0.50) by other therapies) thereby affirming previous data on wound complications in preoperative RT [40]. Moreover, the use of SIB radiation did not lead to high rates of wound complications which was also observed in more recent retrospective data on localized extremity STS comparing sequential boost radiation to SIB radiation [41]. Although preoperative RT causes higher wound complication rates, postoperative RT leads to irreversible fibrosis-related toxicities adversely affecting patients’ limb function, which caused an increasing notion of preferring pre-over postoperative RT among radiation oncologists [22, 40, 42–44].

While the threat of increased acute local RT-related toxicity was not confirmed in our trial, neoadjuvant RCT did correlate with a significant increase in overall acute major toxicity (73.9% vs. 38.9% in “other therapies”, p = 0.019) and hematological toxicity (52.2% vs. 18.5% in “other therapies”, p < 0.001). In the RTOG 9514 trial, a modified MAID regimen with higher ifosfamide dose (2500 mg/m^2^ vs. 2000 mg/m^2^ in the MGH trial) was applied, which caused higher overall and hematological toxicity compared to the MGH trial (e.g., grade 4 leukopenia: 73.4% versus 35.4% in RTOG9514 (28, 35)). Although the present trial applied even higher doses of ifosfamide (3000 mg/m^2^) in the RCT group, grade 3 or 4 hematological toxicity of 52.2% were remarkably lower compared to 91% grade 3 or 4 hematological toxicity in the RTOG 9514 trial. The neoadjuvant RCT did cause higher rates of febrile neutropenia requiring hospitalization compared to the MGH trial (39.1% vs. 6.5% , respectively). However, no treatment-related deaths occurred (28). It is our impression that these differences in toxicity may be attributed to dacarbazine not being administered in our study. Apparently, not using dacarbazine did not negatively affect the outcome and may be the reason for the reduced toxicity when compared to the RTOG 9514 trial (35). Chowdhary et al. also noted a lower complication rate without dacarbazine (43).

Despite the existing toxicity, comprehensive trials investigating histology subtype-specific CTX regimens and an altered number of CTX cycles found three cycles of anthracycline/ifosfamide to remain the best option in terms of oncological outcomes for localized STS with high-risk features [39, 45–48]. Future trials comparing neoadjuvant RCT to RT alone in localized STS with high-risk features should therefore include three cycles of anthracycline/ifosfamide based CTX and standard preoperative RT regimen consisting of 1.8–2.0 Gy/fraction to a total dose of 50 to 50.4 Gy in 25–28 fractions delivered over 5–6 weeks [49].

## Limitations

The current findings should be interpreted in light of the following limitations. Firstly, STS are a highly heterogeneous group of malignant tumors. This is a retrospective study conducted at a single institution and therefore prone to different types of bias (e.g. sampling bias). Moreover, our neoadjuvant RCT cohort’s sample size was relatively small (n = 23, 20% of the entire cohort). In addition, the fact that patients treated with neoadjuvant RCT were fitter, yet had larger median (but not mean) tumor diameters than those who received other therapies, might have interfered with the interpretation of the therapy outcomes of this study. Another important limitation is the heterogeneity of treatment regimens with SIBs in a large proportion of patients and the heterogeneity of the "other therapies" group. However, this does reflect the current diversity of perioperative treatment strategies for STS in everyday clinical setting.

## Conclusions

The current retrospective study found significantly lower LR and DM rates in adult patients with localized G3 STS undergoing neoadjuvant RCT. Nevertheless, the significant increase in major complication rate remains an important concern in the implementation of neoadjuvant RCT as the standard perioperative management of STS. Further prospective and comparative studies are warranted to validate our findings.

## Supplementary Information

**Additional file 1**. **Supplementary table 1.** Univariate and multivariable analysis of OS for G3 sarcoma. n/a (not available).

**Additional file 2**. **Supplementary table 2.** Univariate and multivariable analysis of FFDM for G3 sarcoma. n/a (not available).

## Data Availability

The datasets used and/or analyzed during the current study are available from the corresponding author on reasonable request.

## Abbreviations

BIH: Berlin Institute of Health
CTX: Chemotherapy
DM: Distant metastases
FFDM: Freedom from distant metastases
FNCLCC: Fédération Nationale des Centres de Lutte Contre le Cancer
KPS: Karnofsky Performance Status
LC: Local control
LR: Local recurrence
MAID: Mesna, Adriamycin (doxorubicin), ifosfamide and dacarbazine
MGH: Massachusetts General Hospital
MVA: Multivariable analysis
OS: Overall survival
RCT: Radiochemotherapy
RT: Radiotherapy
RTOG: Radiation Therapy Oncology Group
SIB: Simultaneous integrated boost
STS: Soft tissue sarcoma
UVA: Univariate analysis
