# Supplementary material for: The role of neoadjuvant radiochemotherapy in the management of localized high-grade soft tissue sarcoma
Source: Radiat Oncol. 2022 Aug 8;17:139. doi: 10.1186/s13014-022-02106-2 (PMC9361547; doi:10.1186/s13014-022-02106-2)
Supplement: Supplementary file 1 — Additional file 1. Supplementary table 1. Univariate and multivariable analysis of OS for G3 sarcoma. n/a (not available). [file 13014_2022_2106_MOESM1_ESM.docx]

Supplementary table 1. Univariate and multivariable analysis of OS for G3 sarcoma.

| Variable | G3 sarcoma (N=62) | | | | |
| --- | --- | --- | --- | --- | --- |
|  | **univariate analysis** | | **multivariable analysis** | | |
|  | **HR (95% CI)** | **p-Value** | **HR (95% CI)** | **p-Value** |  |
| Age(in years)  <61  ≥61 | Ref.  1.446 (.617-3.387) | .396 | Ref. |  |  |
| Sex  male  female | Ref.  1.271 (.554-2.915) | .572 | Ref. |  |  |
| KPS  <90  ≥90 | Ref.  .372 (.162-.858) | .020* | Ref.  .617 (.246-1.550) | .304 |  |
| Location  other  extremity | Ref.  .774 (.305-1.963) | .590 | Ref. |  |  |
| Tumor size (in cm)  <8.8  ≥8.8 | Ref.  1.246 (.545-2.852) | .602 | Ref. |  |  |
| Resection margin  R0  R1/2  n/a | Ref.  2.137 (.480-9.512)  1.561 (.362-6.731) | .319  .550 | Ref. |  |  |
| Neoadjuvant R(C)T  Neoadj. RCT  Neoadj. RT alone | Ref.  14.633 (2.831-75.632) | .001* | Ref.  10.234 (1.761-59.469) | .010* |  |

Abbreviation: n/a (not available).
