# Supplementary material for: The role of neoadjuvant radiochemotherapy in the management of localized high-grade soft tissue sarcoma
Source: Radiat Oncol. 2022 Aug 8;17:139. doi: 10.1186/s13014-022-02106-2 (PMC9361547; doi:10.1186/s13014-022-02106-2)
Supplement: Supplementary file 2 — Additional file 2. Supplementary table 2. Univariate and multivariable analysis of FFDM for G3 sarcoma. n/a (not available). [file 13014_2022_2106_MOESM2_ESM.docx]

Supplementary table 2. Univariate and multivariable analysis of FFDM for G3 sarcoma.

| Variable | G3 sarcoma (N=62) | | | | |
| --- | --- | --- | --- | --- | --- |
|  | **univariate analysis** | | **multivariable analysis** | | |
|  | **HR (95% CI)** | **p-Value** | **HR (95% CI)** | **p-Value** |  |
| Age(in years)  <61  ≥61 | Ref.  1.038 (.507-2.125) | .918 | Ref. |  |  |
| Sex  male  female | Ref.  .885 (.406-1.926) | .757 | Ref. |  |  |
| KPS  <90  ≥90 | Ref.  .306 (.143-.652) | .002* | Ref.  .554 (.240-1.277) | .166 |  |
| Location  other  extremity | Ref.  .535 (.250-1.143) | .106 | Ref. |  |  |
| Tumor size (in cm)  <8.8  ≥8.8 | Ref.  1.284 (.609-2.704) | .511 | Ref. |  |  |
| Resection margin  R0  R1/2  n/a | Ref.  1.780(.415-7.644)  1.724(.519-5.727) | .438  .374 | Ref. |  |  |
| Neoadjuvant R(C)T  Neoadj. RCT  Neoadj. RT alone | Ref.  26.677(6.334-112.357) | <.001* | Ref.  16.940 (3.688-79.978) | <.001* |  |

Abbreviation: n/a (not available).
